# The challenges of caring for children who require complex medical care at home: ‘The go between for everyone is the parent and as the parent that’s an awful lot of responsibility’

**DOI:** 10.1111/hex.13092

**Published:** 2020-06-16

**Authors:** Bethan F. Page, Lisa Hinton, Emily Harrop, Charles Vincent

**Affiliations:** ^1^ Department of Experimental Psychology University of Oxford Oxford UK; ^2^ Nuffield Department of Primary Care Health Sciences University of Oxford Oxford UK; ^3^ Helen and Douglas House Oxford University Hospitals NHS Foundation Trust Oxford UK; ^4^ Present address: The Healthcare Improvement Studies Institute (THIS) University of Cambridge Cambridge UK

**Keywords:** caregivers, children, chronic disease, home nursing, medical complexity, paediatrics, parents

## Abstract

**Background:**

Increasing numbers of children with complex health‐care needs are cared for at home by their family. The aim of this qualitative study was to explore the challenges experienced by families caring for children who need complex medical care at home.

**Methods:**

We conducted a thematic analysis of eleven in‐depth interviews with parents who carry out specialist medical procedures (eg, enteral feeding, bowel washouts and tracheostomy care) for their children at home. Participants were purposely selected from an existing sample of interviews with parents whose child had abdominal surgery in the first year of life.

**Results:**

We identified three overarching themes: (a) responsibilities of the parent, (b) impact on daily life and (c) the parent journey over time. Parents have substantial responsibilities, including performing medical procedures, managing emergencies (sometimes life‐threatening), co‐ordinating care and advocating for their child. Their responsibilities have an enormous impact on the family: going out of the home becomes a challenge, there are constant constraints on time, parents are sleep‐deprived and there are wider impacts on siblings. The third theme explores the parent journey over time as parents become experts and make sense of the new normal.

**Discussion:**

The burden of care on families caring for children with complex medical needs is much greater than is generally understood by either multidisciplinary health‐care teams or the general public. Families need to be better prepared and supported for the responsibilities they take on and the burden of care needs to be shared by others.

## 1 INTRODUCTION

Increasing numbers of adults and children with complex health‐care needs are cared for at home, primarily by family members.^1^, ^2^, ^3^, ^4^ As a result of significant advancements in surgery and critical care, many adults and children who may not previously have survived are now cared for at home. Many of these people have on‐going medical needs; amidst growing pressures on health‐care resources, care is increasingly provided by families. It is now common for families to carry out complex procedures such as enteral and parenteral nutrition and tracheostomy care. While care at home brings many benefits, there are significant repercussions for families and the health‐care system that need to be better understood.

Some of the most complex care is provided by parents caring for children with serious chronic conditions.^5^, ^6^ The initial months caring for the child at home can be frightening and overwhelming. There is a lot to learn and many of the medical procedures parents undertake are emotionally demanding.^7^, ^8^ Studies have described the tension some parents feel between their role as a ‘nurse’ and as a parent.^9^ These parents experience significantly more stress than caregivers of healthy children, with increased stress associated with greater parental responsibility for treatment management.^10^ Medical routines, the need for constant vigilance and frequent medical appointments, place significant time demands on parents.^11^ On‐going sleep disturbance is a common problem for these parents and has been found to be associated with poor mental health and relationship difficulties.^12^, ^13^ The burden of care experienced by families can be substantial.^14^

Navigating the complex health‐care system is a challenge for families.^15^ There is often a large multidisciplinary network of professionals involved. This can result in a busy timetable of appointments and problems co‐ordinating care. Despite this large network of professionals, the primary responsibility for managing care lies with parents.^15^ Mainstream health‐care services that parents can readily access, such as General Practioners (GPs), Accident & Emergency (A&E) and Out of Hours services, often lack the expertise to help.^15^, ^16^ This places added responsibility on parents to solve problems themselves, particularly out of hours, when seeking help from specialist staff can be difficult.

Families undertake extensive work, including administering treatments and medications and co‐ordinating care. This work has been described as the ‘burden of treatment’ in the context of self‐care for patients with chronic conditions.^17^, ^18^ In this paper, we use burden of treatment theory as a lens to explore the challenges faced by parents who carry out complex medical procedures. The aim of this qualitative study was to explore the challenges faced by families caring for children who need complex medical care at home. We present a secondary analysis of 11 in‐depth interviews with parents whose children had abdominal surgery and needed on‐going medical procedures at home.

## 2 METHODS

### 2.1 Sample

Participants were purposely selected from an existing sample of 44 in‐depth interviews with parents from the United Kingdom whose child had abdominal surgery in the first year of life.^19^ Extracts from the interviews are available publicly on the Healthtalk.org website.^20^ Parents talked about a broad range of issues such as diagnosis, birth planning, the surgery itself and life back home. Interviews lasted between 1 hour 15 minutes and 3 hours 30 minutes. Further information about the recruitment and participants is available in the original publication and online.^19^, ^20^ Although the original study was designed to explore parents’ experiences of having a baby who needed abdominal surgery, many parents talked extensively about the long‐term impact of caring for their children at home, which has not yet been explored in publications from this data set.^19^, ^21^

The interviews began with unstructured narrative prompted by an open‐ended question and were followed up by a semi‐structured component following up on issues raised by parents and themes suggested by the literature and advisory panel. The open narrative approach to the interviews lends itself to supra analysis, that is secondary analysis of an existing data set to investigate a different question to that of the primary research.^22^ A purposive sample was selected to include families who provided complex medical care for their child. This included medical tasks such as tracheostomy care, feeding tubes, stomas and bowel washouts. A total of 11 relevant interviews were identified and full transcripts accessed through a data sharing agreement. All parents had given informed consent before taking part and consented for their data to be used in publications, stored in an archive, and available (subject to approval) to other researchers for secondary analysis (Berkshire Ethics Committee, 09/H0505/66).

Table 1 shows information about the families. All infants had surgery in their first year of life, but to capture the long‐term impact of this surgery, the original sample included a broad range of ages of the children at the time of interview, ranging from 18 weeks to 9 years (see Table 1). Some of the children remained in hospital following birth for long periods of time, including one child who was in hospital for 3 years before going home. Other children were discharged home shortly after birth and returned to hospital for planned surgery several weeks or months later, with a few readmitted in emergencies.

**Table 1:** Participant demographics and information about the children's needs

Participant identifier	Condition and surgeries	Medical tasks carried out	Child's age at interview	Parent's age at interview	No. of children
01 (Mother)	Exomphalos and auditory neuropathy disorder	Tracheostomy and nasogastric feeding tube	18 mo	34	2
19 (Mother)	Exomphalos and a rare heart disease	Nasogastric feeding tube and Percutaneous endoscopic gastrostomy (PEG)	16 mo	36	3
04 & 05 (Mother & Father)	Exomphalos & tracheomalacia (floppy windpipe)	Oxygen & Jejunostomy	7 y	36 & 34	2
18 (Mother)	Hirschsprung's disease	Bowel washouts	1 y	36	2
28 & 29 (Mother & Father)	Hirschsprung's disease	Bowel washouts	18 wk	35 & 34	2
22 (Mother)	Hirschsprung's disease	Bowel washouts & anal dilations	16 months	31	2
20 & 21 (Mother & Father)	Hirschsprung's disease	Bowel washouts & anal dilations	19 mo	40 & 39	3
39 & 40 (Mother & Father)	Hirschsprung's disease	Bowel washouts and stoma care	5 y	33 & 48	1
24 (Mother)	Congenital diaphragmatic hernia	Tracheostomy, ventilation and gastrostomy	9 y	39	4
14 (Mother)	Diagnosis unknown	Stoma care	6 mo	42	1
35 (Mother)	Blood loss to bowel, necrotising bowel	Total Parenteral Nutrition (TPN)	7 y	41	3

The type of care provided by the parents varied according to the clinical condition. The conditions are listed in Table 1 and further information is given in Appendix S1. Five of the children needed bowel washouts, two had tracheostomies, two had stomas, two needed enteral tube nutrition (nasogastric tube or gastrostomy), one needed home oxygen, one needed parenteral nutrition (feed is delivered into the bloodstream, bypassing the gut) and two needed anal dilations. Appendix S2 explains in more detail what these tasks involve.

### 2.2 Analysis

The first author BP undertook a thematic analysis of the transcripts, using the steps laid out in Ziebland and McPherson's paper on analysing personal experiences of health and illness.^23^ This approach is suitable for analysing secondary data, and ensures all the different issues raised by participants, including negative evidence (‘deviant cases’), are included in the analysis process. The author first read through the interview transcripts noting down potential themes and then coded the interviews with the support of NVIVO v12 (software for qualitative analysis), developing a coding framework iteratively in discussion with LH and CV. The research question was focused on the experiences of parents caring for children at home: parts of the transcript which were irrelevant to the research question (eg, descriptions of the birth) were not coded. The data were organized under anticipated and emergent themes, used constant comparison techniques to group the data. The codes were revised through an iterative process. For each theme, a ‘one sheet of paper’ (OSOP) analysis was conducted.^23^ Summaries of each code were then written and re‐written and discussed with the research team. Each code was illustrated with quotes.

## 3 RESULTS

Parents described facing a number of challenges when caring for children with complex medical needs. The first theme explores parents’ responsibilities, the second looks at the impact on daily life and the third explores the parent journey over time. Table 2 shows illustrative quotes for each theme to give an overall sense of the coding structure and challenges faced by families.

**Table 2:** The themes and subthemes with illustrative quotes

The responsibilities of the parent	Performing medical procedures	*You try pinning a 14 mo old and jamming a [nasogastric] tube down her nose, it's not nice for anybody especially when they can scream at you and the more she screams the less it will go down*. (ID19)
	Managing emergencies and problems	*His body was all limp and we couldn't wake him and he was completely unresponsive and we knew that he was like being poisoned and at that point we were like we have to do a flush out immediately we don't even have time to get him to hospital*. (ID39&40)
	Co‐ordinating care	*The go between for everyone is the parent and as the parent that's an awful lot of responsibility and an awful lot of information you have to remember*. (ID19)
	Advocating for your child and fighting the system	*We still have someone in overnight we get five nights a week [um] which we have to fight for on a regular basis which is the odd thing because we had a fight to get him out of hospital and then six months down the track they were trying to reduce his, his package having told me, you know, for three years I can't take him home because he needs someone overnight. (ID24*)
Impact on daily life	Constraints on time	*My GP has said ‘Would you like some counselling?’ and in the same breath he said ‘Can you fit it in? it's just going to be another appointment to you?’ You're right I haven't got time to talk to anyone about how I feel because I’m too busy looking after my kids and their hospital appointments, I just don't have time*. (ID19)
	The challenges of going out	*And I thought ‘How can we go anywhere, we've got suction pumps, we've got oxygen tanks, we've got, more medical stuff for feeding things than you can shake a stick at.’ If we went anywhere we had to have three pushchairs and four adults for one baby to carry everything, so we just didn't go out*. (ID19)
	Lack of sleep	*And over time, just the exhaustion of trying to [um] look after him just got increasingly tough and unmanageable and, actually, to the point of being quite dangerous because, obviously, you have to be awake and alert and ready to change his [tracheostomy] tube or give him suction at any time*. (ID01)
	Impact on other siblings	*I go upstairs to say goodnight to him [other sibling] …he's already fast asleep and I think yeah, and then I go downstairs and spend another half an hour with [daughter] doing all her procedures and think yeah he missed, he missed us yeah*. (ID35)
The parent journey over time	Becoming an expert	*So by doing it for a week at home even, where it's just you, you are actually becoming more experienced and better at doing it than the staff just by the number of times you do it. And then when you get to a stage where you've been doing that for two months, you know, you are the best person who could possibly do it*. (ID28&29)
	The new normal	*It's been really tough on family life but equally we've had days were we've sat in the back garden and everyone's been out and we've gone ‘Ooh look for five minutes we're just a normal family’ and then [Name] will throw up and [Name] will dislocate her shoulder [laughs] [Name] will scream because she's too tired*. (ID19)

### 3.1 The responsibilities of the parent

Parents had numerous responsibilities beyond those most parents have for their child. These included performing medical procedures, managing emergencies, co‐ordinating care and advocating for their child. These responsibilities would traditionally be held by health‐care providers, who in comparison with parents, receive far more training and on‐going supervision. The sense of responsibility parents felt was particularly acute in the period following discharge from hospital, when parental confidence was still building and routines were unfamiliar. For some parents, there was a real sense of being alone in these responsibilities.

#### 3.1.1 Performing medical procedures

Table 3 details some of the descriptions parents gave about the medical procedures they perform for their child. Many tasks were technically and emotionally demanding for parents especially in the early days of caring for their child. This was particularly true for procedures which were perceived to be painful or distressing for their child. Some parents rationalized this by thinking about the long‐term gains.

I don’t think I could do the dilations that was [Partner]’s job to do… he [child] used to scream when we first started doing them, it was a horrendous scream and I would sort of have to go out of the room I couldn’t, I couldn’t bear it and, but you do just have to switch off and you’re doing it so it that in the future he’s, he’s well. (ID20&21)

**Table 3:** Parents descriptions of the medical procedures and tasks they do for their children

Passing a nasogastric tube	*And the first time I pulled the NG tube out was in the middle of the night in the dark two days after we brought her home… it was awful my husband pinned her head down I jammed the tube in hoping that I hadn't hit her lungs and that I’d hit her stomach*. (ID19)
Dilations	*We had to use a dilator with her every day which is like a metal thing that you poke up her bottom to make sure it doesn't shrink but it did shrink. So then we had to and again the worst possible outcome there is more surgery to stretch the bowels. So [um] yeah we had to use a bigger dilator and do it more often and those things were really, really awful*. (ID18)
Bowel washouts	*Oh a special kind of horror really… we would do his wash out and the wash out can take anywhere from about 20 min to an hour… You do a wash out at 10:00 and one at 6:00… And sometimes even three times a day if he particularly distended, you know… it's definitely a two man job…I think doing it that way was way preferable to him being in hospital I absolutely, I mean it was a pain… it's not an easy thing, it had to be said*. (ID28&29)
Stoma care	*You're afraid of kind of tearing the wound or hurting something and then the stoma would bleed and you kind of have to get used to the fact that it's bleeding*. (ID14)
Total parenteral nutrition	*We've also had times where, you know the giving set has broken so, you know she's, the TPN is just leaking into the bed and she, you know, her central line is just essentially open to the air, you know, she could bleed out, she could get air in her line*. (ID35).
Medication management	*She was having several different tablets or.. it wasn't tablets, it was all liquids but medications a day and at lots of different intervals… and that was always quite stressful organising that*. (ID04&05)
Medical decision making	*It was a case of getting through every day and every time we changed his nappy, sort of be a wet nappy, you know, prodding his stomach and seeing if it was hard or soft, does it look big. Got so many pictures of him on my phone throughout the day just to see whether or not his stomach looks big*. (ID20&21)

The procedures parents had to carry out had been done by nurses and doctors in hospital. One parent commented that ‘*you are basically just being a nurse*’ (ID04&05). Some had been trained how to perform medical procedures in hospital as part of their preparation for going home, whereas others had limited experience. Most were daunted by the sense of responsibility when they first did the tasks at home, without the back up of health‐care professionals.

Suddenly we had to do it all… when neither of us have barely any medical training whatsoever… I was confident doing it in the hospital but its’s different being confident doing it an environment where you have lots of medical professionals to help you if you get stuck, to doing it by yourself at home, alone. (ID19)

Some felt they had not received enough training and some of the problems they experienced could have been avoided with more preparation before going home.

We didn’t know what we were doing… we felt a bit out of our depths… it was a lot of trial and error with the [stoma] bags as well [um] a lot of leaks… a lot of getting up in the night and having to get him in the shower and wash him down and looking back on it that was unnecessary… if we had had the support, the correct training and support. (ID39&40)

#### 3.1.2 Managing emergencies

One of the most psychological challenging responsibilities parents faced was managing emergencies. Some parents had to manage life and death situations at home, including one parent who was responsible for administering home oxygen when her child deteriorated.

She [child] would go mottled and she’d go blue and she’d whimper and then … her oxygen levels would drop [um] and then her peripheral pulses would go [um] and she’d just lie and whimper [um] hence we had the home oxygen. (ID19)

All parents were responsible for making decisions on when to seek help from medical professionals. Some were worried they might not detect problems and call for help quickly enough, including one parent who described lasting trauma from an incident which led to her child being resuscitated.

By the time we got to the hospital [name of child] was so shut down they could only find a vein in her head [um] and she had to be resuscitated… she was so close to dying and I hadn’t realised how close to dying she was … that was the single worst time of everything the whole lot and that’s the memory you have when you’re lying in bed thinking about your mind works overtime, that memory there, that makes me stop in my tracks. (ID19)

Decisions on when to seek help are complicated by the fact that General Practitioners (GPs), Accident & Emergency (A&E) and out of hours/after hours care often lack the specialist knowledge required to advise parents on how to treat these children. Some parents felt they should have received better preparation for dealing with problems and emergencies themselves.

#### 3.1.3 Co‐ordinating care

Parents are responsible for co‐ordinating all aspects of their child's care. Some families had a complex web of health‐care professionals involved, with one parent (ID19) noting that they were ‘*21 consultants’* involved in her child's care. Parents become the only one who understood the complex ‘map’ and become the communication link between the professionals.

So a while back I drew like a mind map of all the professionals who are involved in [son]’s care and everything and [um] if, if needs be, I can show that to people. Actually, it’s quite a powerful way of demonstrating the complexity of, of what you have to deal with. (ID01)

Families had numerous appointments and visits to manage, with some having 2‐4 appointments each week. Arranging, prioritising and attending all these appointments was time consuming and brought with them a multitude of logistical challenges.

Parents were also responsible for complex medication regimes. Some parents experienced difficulties getting prescriptions for rare and specialist drugs and felt they had to advise GPs and make decisions about medication changes themselves.

I had to frogmarch her in to the surgery one day and tell the doctor I wanted ranitidine because she had silent reflux, you know, my, my responsibility, my decision, just give me the medicine. And this poor young doctor looked petrified but he gave me the prescription and it worked and thank god because it was, I guess it was a bit of a guess on my part as well. (ID18)

As well as medication management, parents were also responsible for managing supplies and regular deliveries, and there were often hiccups. Homes became medical spaces, storing these vital supplies.

We were having loads of trouble with the supplier, they were throwing our supplies over the back gate when it was raining… So the boxes were getting wet and I was calling them and I was like ‘I can’t use stoma bags that have been outside in the rain you’re gonna have to re‐send them’ (ID39&40)

#### 3.1.4 Advocating for your child and fighting the system

Parents advocated strongly for what they felt was best for their child and family. Parents wanted to feel they were able to make decisions and sometimes got frustrated when they felt the rules and regulations were being applied without flexibility. In some cases, there were disagreements between parents and health‐care professionals. For example in one instance, health‐care professionals threatened to report the parents to social services citing safeguarding concerns because the parents wanted to care for their child at home rather than in hospital.

We said well this is ridiculous we’re not doing this and they said well at that, at this point then that becomes a child protection issue we’re gonna have to bring in Social Services because you’re refusing medical care for your child. (ID28&29)

Parents were their child's advocates in other aspects of life, such as education. Some felt like they were constantly fighting. If they disagreed with a decision made by a health‐care professional or another authority, they were threatened. The constant fighting had serious psychological impact on some parents.

We received a letter in the post saying we’re reducing your [care] package to three nights… If you don’t agree with what they’re doing, if you say no to anything then they just, they, you know, threaten to take your package away and bully you until you do what they want. So we’ve actually have the MP involved now to see whether, you know, she can sort it out because at the time when they did that with the bed I ended up having panic attacks and I [um] I was really, really, really distressed, you know. (ID24)

### 3.2 The impact on the family and day‐to‐day life

The impact on day‐to‐day life for families was substantial. There was not enough time to do everything that needed doing, going out the house became difficult and for some, sleep‐deprivation became a common feature of life. The care needs of the child placed constraints on many aspects of life, and impacts were felt on the whole family. Parents were particularly concerned about the impact on other siblings.

#### 3.2.1 Constraints on time

Lack of time was a dominant feature of parents’ narratives. There was simply too much to do: arranging and attending appointments, managing equipment deliveries, looking after other siblings, washing clothes and bedsheets for children with incontinence and finding time to do household chores.

I seemed to be very busy like [um] she did go through tons and tons of clothes obviously because they all got leaked with bowel fluid all the time… so I was washing her clothes, drying her clothes, sorting stuff out, you know, waiting for the next doctor, waiting for the next nurse, waiting for hand over, changing her nappies, getting the nappies weighed,… I don’t know where the time goes but you are like incredibly busy. (ID35)

Over time parents developed routines. Some felt others (eg, health‐care professionals, friends and those who commission or manage health‐care services) did not realize just how much they had to juggle and how little time they had. Parents often had no time for themselves.

‘There has been days where I, where I’ve gone ‘Enough. If I don’t leave this house for five minutes and go and get some fresh air I’ll actually not come back’ because there’s so much in your head and the times where you do have five minutes to sit down and relax you’re on the exomphalos [condition affecting the abdominal wall, see Appendix S1] support group checking up on other people’s kids… so the time when you do have as you’re down time, my respite care, I spend most of my respite care running to and from school dealing with my other children’. (ID19)

#### 3.2.2 The challenges of going out

Families were unable to leave the house and go out with their child after coming home from hospital. There were practical constraints such as being unable to carry all the medical equipment or not being able to take their child out due to infection risk, and psychological reasons for not going out, especially soon after coming home from hospital.

I was afraid to take him out for long periods of time because I didn’t want to have to do a nappy change or a stoma bag change in a public place. Not just because it is so messy but also I didn’t want other people to kind of be able to see me doing a stoma bag change. (ID14)

Over time going out became easier, although life had to be fitted around time‐specific medical procedures, and practical challenges, such as worries about the risk of infection, remained. Participant 35 described the detailed planning required to make activities possible, such as going swimming or on a family holiday. For some, it was important to stay near to their specialist hospital in case there was a problem.

Some felt they could not go anywhere without their child, as it was too much responsibility to leave them with a relative or a friend. Others felt confident occasionally leaving their child with a trusted family member, but they did not go far in case there was a problem.

We don’t leave her [daughter] at all when she’s connected so we can’t go out in the evenings when she’s on an infusion just because it’s too difficult and it’s too much responsibility really for other people [um] and like we say we’ve had all of these different things go wrong over the years and although they’re rare… they’re quite life threatening. (ID35)

#### 3.2.3 Lack of sleep

Lack of sleep was a real struggle for some parents, especially soon after coming home. Some children had on‐going medical needs that needed attending to at night, such as giving feeds and medications, or responding to equipment alarms. There was a lot to do even at night‐time. Parents were constantly alert. One parent described sleeping with the light on sitting up (ID04&05).

The children with tracheostomies had care packages which provided paid carers for a set number of hours a week. Not all parents were able to trust the carers enough to sleep: after an error made by a carer, one parent fitted a video camera to monitor overnight care.

I said I’m sorry I cannot go to bed and leave [son] down here with him [the paid carer] all the time cos when, on the nights that he was here as well we’d have a video camera in there and I didn’t sleep because I was so worried. (ID24)

Lack of sleep had a wider impact on parents, such as on their relationship with their partner and their children. One parent (ID24) was prescribed antidepressants to help her sleep. A few remarked on the dangers of performing medical tasks when they were so tired.

On one tube change occasion, I put in a smaller [tracheostomy] tube rather than the right tube size and didn’t notice for a couple of hours [um] and I thought then, this is actually dangerous. I can’t do this anymore. (ID01)

#### 3.2.4 Impact on siblings

Concern for the impact on other siblings was a key feature of parents’ narratives. They felt guilty and torn: they had to prioritize the needs of their medically complex child, and didn't have enough time to spend with their other children.

They [younger siblings] were screaming because they wanted feeding but I had to deal with [son] because otherwise he, you know he’d be coughing and he’d vomit and then he wouldn’t he’d drop his oxygen and then he, you know, wouldn’t be able to breathe so again it’s all, when it’s always been where you’ve had to pick [son] over the other kids. (ID24)

Most parents reflected that things had been hard for the siblings, for example feeling other siblings had grown up quickly, or played up for attention. Parents were concerned about siblings watching distressing procedures, and long‐term trauma, as well as the impact their own mental health and lack of sleep was having on their children.

I know my teenage daughter definitely suffers from it [PTSD] cos she can’t stand to look at [Name]’s tubes, she can’t stand to be in the room when the children are crying. (ID19)

Parents did what they could to help their other children to cope, for example some tried explaining the medical procedures to their children to help them understand what was happening. Others described strategies for keeping their other children busy so they could concentrate when they did procedures.

She [sibling] has seen the, the dilator and I’ve explained it’s to make [Baby 2]’s bum well and [um] cos she’s at that stage of ‘What’s that’ and ‘What’s happening’ and ‘Why’s that, what’s that, why’s [Baby 2] crying’ and things and so, you know, we’ve just, just explained it as well, best we can. (ID20&21)

### 3.3 The parents’ journey over time

Parents invested a lot of time and effort in developing substantial expertise in the medical needs of their children and felt that aspects of their expertise exceeded that of clinicians. Parents were on a journey to find a new normal for their family as they tried to make sense of their new reality which few others understood.

#### 3.3.1 Developing expertise

Parents became experts in their child's medical needs by doing procedures daily, learning through trial and error, and doing extensive research. They used a range of sources, including online information, advice and recommendations from their peers, and journal articles. Over time, many developed more expertise than the professionals. They wanted their expertise to be valued by professionals.

Don’t undervalue the amount of time and energy [um] that parents put into trying to find out as much as they can and also knowing their child inside out and back to front. (ID01)

Parents developed expertise in managing and responding to problems, and detecting early warning signs and became increasingly confident in medical decision making.

As parents developed expertise, some also became very wary of the care given to their child in hospital or by other professionals and carers. For example, one family (ID28&29) describe the distress caused to their child during a bowel washout performed in hospital.

It was absolutely brutal…clearly someone who is mostly used to doing things on unconscious people, you know, and he [child] was hysterical in the end. (ID28&29)

#### 3.3.2 The new normal

Parents were on a journey to find a new normal as they adapted their lives. The pursuit of normality was a key feature of their narratives. Some families talked about normal life returning, in relation to their child's condition getting better or being able to go out and do everyday things. Feeling like their child was ‘normal’ was important to some: they made references to ‘normal’ activities their children (or other children with the same condition) do, like playing football. Others reflected that their normal wasn't necessarily what others saw as normal, but something they had come to accept.

He’ll be fine, it will be fine, they’re just a different normal. (ID20&21)

Parents reflected on the differences between their family and others. For example, not being able to go out occasionally and leave their child with someone else, like ‘*normal married couples*’ (ID39&40). Family roles changed as families adapted to a new normal.

Yeah he [husband] does team well and I do team ill that’s very much the balance now, he gets all the kids who are well that day and goes out and does stuff and I keep the kids who are ill and need medical attention. (ID19)

For some parents, chronic stress was an enduring part of their new normal.

It’s like because you’re living on the edge all the time, the littlest curve ball that is thrown into the mix just sends you over the edge. (ID24)

Some parents felt that they weren't told about the realities of caring for a child with complex medical needs, or that the picture that was portrayed to them doesn't match reality. Many felt that health‐care professionals, commissioners and friends don't understand their reality.

We knew that we would have a lot of obstacles to face…I did not anticipate our life to become completely and utterly consumed by medical needs. (ID19)

## 4 DISCUSSION

The analysis reported in this paper provides valuable insights into parents’ experiences of caring for children who need complex medical procedures at home. Parents take on substantial responsibilities well beyond those most parents have for their children. They have to learn to do technical and distressing medical procedures, manage emergencies and co‐ordinate their child's care. They become advocates so they can navigate and fight the complex health and care system. Their responsibilities have an enormous impact on the family. There is often too much to do and not enough time. Some parents become chronically sleep deprived and engaging in normal activities is very difficult. However, over time parents become experts in their children's medical needs and adapt their lives to a new normal.

### 4.1 Burden of treatment

Burden of treatment theory was originally developed to describe the work of patients with chronic conditions (and their caregivers) who are increasingly delegated tasks previously undertaken by the health‐care system, such as self‐monitoring, drug management and passing information between health‐care professionals.^17^, ^18^, ^24^ The parenting of children with complex medical needs is similarly complex and burdensome and has been described in one study as ‘intense parenting’.^14^ The capacity of families to take on these responsibilities is dependent on many factors such as the medical complexity of the child and the psychosocial complexity of the family.^25^ An additional challenge for parents of children with the most complex needs is that hardly anyone else has the expertise to care for their children. This contributes to a substantial sense of responsibility and means that parents are isolated and cut off from normal support networks. Parents are taking on significant responsibilities, many of which are within the remit of highly trained professionals in hospital settings, but given comparatively little support and preparation.

Parents learn to navigate the complex health and care system and become advocates for their children. As other studies have found, the burden of navigating the system can have a negative impact on parents’ mental health.^25^ May and colleagues described burden of treatment as the ability to exploit opportunities to utilize health‐care service. Parents in this study similarly had to develop structural resilience (potential to absorb adversity), social capital (informational and material resources), social skills (securing co‐operation) and functional performance (potential to do the work).^24^ The health and care system may sometimes appear to try to control parents (eg, by involving social services or threatening to take away a care package as was the case in the interviews) which can lead parents to feel they are ‘fighting the system’. Studies from other countries have also found that parents feel they are fighting the system.^26^, ^27^ Systems, and the rules and regulations which govern them, are often created around services, rather than designed to meet the needs of children and families. Co‐ordinating care and advocating for their child becomes a time consuming endeavour for parents and adds to the burden of care.

### 4.2 Support for parents

Families have substantial expertise that needs to be valued and listened to,^21^ but they need help to ease the burden of care, and more preparation and support for the responsibilities they undertake. There is a clear psychological impact on the whole family which needs to be core to the preparation and support parents receive. There are existing resources from the literature on trauma‐informed care which may help families cope with managing distressing procedures and emergencies at home.^28^ Online education materials are available at HealthCareToolBox.org. Some of parents’ existing responsibilities, for example co‐ordinating care and navigating the system, ought to be provided by statutory services. Although this is mandated by clinical guidelines in the UK,^29^ it is difficult to achieve in practice. It is well recognized that greater integration between health‐care services has the potential to help with co‐ordination of care.^25^, ^30^ In Canada and the United States, models of care for children with medical complexity are being tested in which care co‐ordination is provided.^31^ In the UK, community paediatricians and palliative care often help with co‐ordination of care and the charity Well Child funds nurses who help with co‐ordinating care, day‐to‐day emotional and practical support and also help train parents and carers, but these services need expanding.^32^ In order for parents to feel safe leaving their child with others (eg, paid carers, family members, respite services), high‐quality training needs to be more readily available. There is evidence that greater access to respite care, peer support and financial aid can also help reduce the burden on families.^33^, ^34^ Cuts to services which support families (eg, respite care) are creating more acute problems and high costs in the longer term when families are unable to cope.^35^

### 4.3 Strengths and limitations

The open narrative approach to the interviews enabled the researchers to uncover rich insights into the experiences of parents caring for children who require complex medical care at home, despite the fact that the interviews were originally conducted to answer a different research question. Some of the findings clearly resonate with themes identified in other qualitative studies, for example feeling you are fighting the system and parents’ experiences of doing distressing procedures.^8^, ^14^, ^26^ Parents of children with complex needs have limited time and can be difficult to recruit; by conducting secondary analysis of existing interviews we have maximized the utility of participants' data.^36^ The analysis was conducted primarily by the first author, a psychologist who is conducting research on support for parents of children with medical complexity. The author's extensive knowledge of the subject area meant they were well‐placed to identify anticipated themes to enrich the analysis, as well as emergent themes from the data. The analysis was overseen by the second author, an experienced social scientist and qualitative researcher who conducted the original interviews and the third author, a paediatrician. This meant that the analysis could be checked firstly against the insights from the original interviewer from ‘being there’, and secondly against the experiences of a clinician who works with children with medical complexity who confirmed the analysis ‘rung true’ with the experiences of families she supports. The sample covered a wide range of on‐going needs and medical procedures so the findings may transfer to children with other conditions, beyond abdominal surgery which was common to all the children in our sample.^37^ The range of ages of the children at the time of interview meant that we captured parents’ experiences at different time points, but conversely meant that not all participants could reflect on the long‐term impacts.

## 5 CONCLUSIONS

Children with serious clinical conditions of many different kinds have benefitted from advances in surgery and critical care.^38^, ^39^ Little is known about the longer term outcomes for these families, and the day‐to‐day of caring for children with on‐going complex medical needs at home. This study highlights the challenges families are facing. The responsibilities placed on parents are substantial. Parents need to be better prepared and supported to take on these roles, whether that be in managing emergencies, performing medical procedures or co‐ordinating care. There is scope for some of these responsibilities to be shouldered by the health and care system. Increased access to properly funded respite care, care packages that parents trust, and dedicated professionals to help parents navigate the complex health‐care system could all help reduce the burden.

## CONFLICTS OF INTEREST

The authors declare that they have no conflicts of interest.

## AUTHOR CONTRIBUTIONS

BP analysed the data with input from LH and CV. The original interviews were conducted by LH. BP drafted the paper, and all authors commented and contributed to the revisions. All authors read and approved the final version.

## ETHICAL APPROVAL

Berkshire Ethics Committee, 09/H0505/66. All parents gave informed consent before taking part and consented for their data to be used in publications.

## Supporting information

Supplementary Material

Supplementary Material

## Data Availability

This paper is based on the analysis of qualitative interviews. Interview transcripts may be made available for secondary analysis by researchers through entering into a data sharing agreement with the University of Oxford. Extracts from the interviews are available on the Healthtalk.org website.
