# Supplementary material for: The challenges of caring for children who require complex medical care at home: ‘The go between for everyone is the parent and as the parent that’s an awful lot of responsibility’
Source: Health Expect. 2020 Jun 16;23(5):1144–54. doi: 10.1111/hex.13092 (PMC7696130; doi:10.1111/hex.13092)
Supplement: Supplementary file 1 — Supplementary Material [file HEX-23-1144-s001.docx]

**APPENDIX A: Further information about the conditions of the children**

*Exomphalos*

Three of the children had exomphalos, which is an abdominal wall defect where some of the intestines and other organs develop outside the abdominal cavity during the pregnancy. The corrective surgery was to return the intestines to the abdominal cavity. These children tended to have the most complex needs of the sample. Following their corrective surgery, the children had varying levels of ongoing needs. All three of the children with exomphalos needed enteral feeding tubes (of various types) and two needed some level of respiratory support (one had a tracheostomy and another needed home oxygen for emergencies).

*Hirschsprung’s disease*

Five of the children had Hirschsprung’s disease which is a rare disorder of the bowel, where the nerve cells do not develop all of the way down the bowel. The section of bowel with no nerve cells cannot relax and it can lead to a blockage. Children with Hirschsprung’s need surgery to correct this, which typically happens in the first few months of life when they are strong enough for the surgery. Some children also need stomas. The children in the interviews with Hirschsprung’s disease all needed bowel washouts at home for a few weeks or months before the corrective surgery, with some continuing to need them occasionally post-surgery. One child also needed anal dilations and another had a long term stoma.

*Congenital diaphragmatic hernia*

One child (ID24) had a congenital diaphragmatic hernia which is a defect in the diaphragm muscle, the muscle that separates the chest and abdomen. As a result, the contents of the abdomen (stomach, intestines and/or liver) grow into the chest cavity in utero which can affect the development of the lungs. This child had continuing complex needs with a tracheostomy and feeding tube (gastrostomy).

*Undiagnosed conditions*

Two of the children had undiagnosed problems which required abdominal surgery. One of these children (ID35) was born healthy but at 10 weeks old became unwell very quickly – experiencing convulsions and turning blue. After several days doctors and surgeons identified that her bowel was necrotising and performed emergency surgery to remove the section of infected bowel. The child was left dependent on total parenteral nutrition (TPN) through a central line. The other child (ID14), whose diagnosis remains unknown, had a stoma fitted in an emergency at a few days old but has few ongoing needs now.
