# Supplementary material for: The challenges of caring for children who require complex medical care at home: ‘The go between for everyone is the parent and as the parent that’s an awful lot of responsibility’
Source: Health Expect. 2020 Jun 16;23(5):1144–54. doi: 10.1111/hex.13092 (PMC7696130; doi:10.1111/hex.13092)
Supplement: Supplementary file 2 — Supplementary Material [file HEX-23-1144-s002.docx]

**APPENDIX B: Descriptions of the tasks carried out by the parents in the interviews**

**Enteral tube feeding (feeding through the nose, stomach or bowel)**

*Nasogastric feeding tubes:*

Nasogastric (NG) feeding is where a narrow tube is inserted through the nose down into the stomach. The tube is used to give fluids, medications and liquid food directly into the stomach. Family carers give feeds and/or medications to their child through the NG tube. Some family carers also insert new tubes themselves. Parents need to pH test the tube (to avoid feeding in to the lungs if the tube is misplaced).

*Gastrostomy feeding (e.g. PEG, button):*

Children with a gastrostomy are fed directly into their stomach. A gastrostomy is a surgical opening through the abdomen into the stomach. A feeding device is inserted into this opening. There are many types of devices that children can have including a PEG tube (Percutaneous Endoscopic Gastrostomy) and a low-profile button device such as a MINI or MIC-Key. Family carers give feeds and/or medications, and also care for the gastrostomy site and the skin around it. Some of the devices such as buttons need be changed regularly and can also be pulled out by accident. Some family carers insert new buttons themselves.

*Jejunostomy feeding:*

A jejunostomy is a surgical opening into the beginning of the small intestine (the jejunum) which is just below the stomach. Similar to a gastrostomy, a tube is inserted into the opening. The care is very similar to gastrostomy feeding, described above. Good standards of hygiene are even more essential as the tube bypasses the anti-infection mechanisms which are present in the stomach.

**Total parenteral nutrition**

In total parenteral nutrition feeds/fluids are administered directly into the bloodstream (usually through a large vein in the chest area), rather than through the gastrointestinal tract (stomach or bowel) like in enteral feeding. A central line or peripherally-inserted central catheter (PICC) line is inserted and feeds are given through a port. TPN is higher risk than enteral feeding. Strict aseptic procedures have to be followed when connecting feeds or touching the site as there are risks of serious infections and sepsis.

**Tracheostomy care**

A tracheostomy is an artificial opening into the windpipe (trachea) which is held open by a tracheostomy tube. It helps children to breathe. Air goes in and out through the tracheostomy tube and bypasses the nose and mouth. Family carers who care for a child with a tracheostomy learn to perform various tasks, this includes: removing excess secretions by suctioning, changing the tapes which hold the tracheostomy tube in place, caring for the skin around the stoma site and organising equipment and supplies. Some children who have tracheostomies may also need the help of a ventilator (invasive ventilation).

**Nasal cannula delivered oxygen**

Oxygen therapy involves breathing in air from a machine which contains more oxygen than normal air. Nasal-cannula therapy is common way of delivering oxygen. The oxygen is administered through a tube that sits at the opening of the nose.

**Bowel washouts**

A thin tube is inserted into the child’s bottom and filled with a warm saltwater solution. This softens the faeces and flushes it from the child’s bowel.

**Anal dilations**

If a child has had a new anus created or widened, in some cases, parents are asked to stretch (dilate) it using a probe called a dilator. Anal dilation is needed to keep the child’s anus open to the right size. This is typically done once or twice a day. Dilations are continued until the rectum has completely healed and has reached a desired size.

**Stoma care**

Some children need a stoma which is an artificial opening for the bowel. Faeces go into an external pouch called a stoma bag. Stoma bags have to be emptied and changed several times a day.
